# Neuroimaging and plasma biomarker differences and commonalities in Lewy body dementia subtypes

**DOI:** 10.1002/alz.70274

**Published:** 2025-05-22

**Authors:** Naomi Hannaway, Angeliki Zarkali, Rohan Bhome, Ivelina Dobreva, George E. C. Thomas, Elena Veleva, Irene Gorostiaga Belio, Katie Tucker, Amanda Heslegrave, Henrik Zetterberg, Rimona S. Weil

**Affiliations:** ^1^ Dementia Research Centre UCL Institute of Neurology London UK; ^2^ UCL Hawkes Institute Department of Computer Science London UK; ^3^ United Kingdom Dementia Research Institute UCL London UK; ^4^ Department of Neurodegenerative Disease UCL Institute of Neurology London UK; ^5^ Department of Psychiatry and Neurochemistry Göteborgs Universitet Mölndal Sweden; ^6^ Department of Neurology Sahlgrenska University Hospital Mölndal Sweden; ^7^ Wellcome Centre for Human Neuroimaging UCL London UK; ^8^ Movement Disorders Centre, UCL London UK

**Keywords:** biomarkers, imaging, Lewy body dementia, MRI, Parkinson's disease dementia, plasma

## Abstract

**INTRODUCTION:**

Despite ongoing debate about whether Parkinson's disease (PD) dementia (PDD) and dementia with Lewy bodies (DLB) are separable diseases or a single Lewy body dementia (LBD) spectrum, there are limited investigations of differences between these conditions.

**METHODS:**

We used fixel‐based diffusion magnetic resonance imaging and plasma measures to examine white matter integrity and burden of amyloid pathology (using phosphorylated tau‐217 [p‐tau217]) in 47 patients with DLB, 21 with PDD, 29 with PD, and 23 age‐matched controls.

**RESULTS:**

We show reduced fiber cross‐section in LBD versus PD, and increased concentrations of plasma neurofilament light chain and p‐tau217; with p‐tau217 and fiber cross‐section associated with cognition. Fiber density was reduced in PDD versus DLB, but neither plasma measures nor fiber cross‐section differed between LBD subtypes.

**DISCUSSION:**

Our findings suggest that the presence of dementia in LBD is associated with poorer white matter macrostructure and may relate to pathological protein accumulation. Conversely, differences between DLB and PDD may be driven by other factors.

**Highlights:**

- Plasma neurofilament light and phosphorylated tau‐217 were increased in Lewy body dementia (LBD) relative to Parkinson's disease (PD) and controls.
- Magnetic resonance imaging (MRI) white matter macrostructure (fiber cross‐section) was reduced in LBD relative to PD.
- In contrast, MRI white matter microstructure (fiber density) was reduced in PD dementia compared to dementia with Lewy bodies.
- Differences between dementia with Lewy bodies and PD dementia were distinct compared with those between LBD and Parkinson's with normal cognition.
- Our findings suggest that dementia with Lewy bodies and Parkinson's dementia differ in underlying processes distinct from those driving dementia.

## 1 BACKGROUND

Lewy body dementia (LBD), comprising Parkinson's disease dementia (PDD) and dementia with Lewy bodies (DLB), is the second most common degenerative cause of dementia.^1^ PDD and DLB are characterized by overlapping symptoms of dementia, motor parkinsonism, hallucinations, cognitive fluctuations, and rapid eye movement sleep behavior disorder.^2^ They share overlapping pathologies, including cortical build‐up of alpha‐synuclein, amyloid beta plaques, and tau tangles.^3^, ^4^ Currently DLB and PDD are defined clinically, based on the “1‐year rule.” If dementia precedes or develops within 1 year after motor symptoms, DLB is diagnosed, whereas if dementia occurs more than 1 year from onset of motor parkinsonism, PDD is diagnosed. There is continued debate about whether DLB and PDD form a single disease spectrum,^5^, ^6^ or are two separable diseases.^7^ However, few studies directly compare these patient groups.

Complementary information from fluid biomarkers and advanced brain imaging techniques offer the potential to understand disease‐related changes in LBD. Diffusion‐weighted imaging (DWI) is sensitive to changes in white matter connections, reflecting axonal damage that occurs before gray matter atrophy.^8^ Measured using DWI, fractional anisotropy (FA) is reduced in LBD in regions including the corpus callosum, superior longitudinal fasciculus, and parietal lobes.^9^, ^10^, ^11^, ^12^ Comparisons between LBD subtypes are less clear. Although some studies found no differences,^11^ others observed widespread changes in DLB versus PDD, with reduced FA in visual association, posterior temporal, and posterior cingulate.^12^

However, conventional DWI has limited sensitivity in regions with crossing fibers, which are present in most of the brain.^13^ Higher‐order diffusion models, such as fixel‐based analysis, overcome this by examining individual fiber populations within a voxel,^14^ increasing sensitivity in regions with crossing fibers. We previously showed that PD at high versus low risk for dementia have widespread white matter changes, with greater loss of fiber cross‐section associated with poorer cognition after 18 months.^15^ Whether these differences are seen between LBD subtypes is not yet established.

Widening availability of fluid markers enables the collection of complementary information about pathological accumulations and other disease markers. For example, neurofilament light chain (NfL) is a marker of neuronal injury,^16^ reflecting axonal damage.^17^ Increased plasma NfL is seen in DLB relative to controls^18^ and PDD compared to PD,^19^ with higher concentrations associated with poorer cognition^19^ and white‐matter loss.^8^ Co‐pathology is frequently seen in LBD, with amyloid beta and tau the most common pathological accumulations.^3^ These can be detected with plasma tau phosphorylated at threonine 181 (p‐tau181) and 217 (p‐tau217), which are both highly sensitive to brain amyloid beta and tau, and established as markers of amyloid beta and tau pathology.^20^, ^21^ Plasma p‐tau181 is increased in DLB compared with controls,^18^, ^22^ and associated with poorer cognition.^22^ In PDD, the results are less consistent, with no differences between PD patients with and without dementia.^19^ However, p‐tau217 has not yet been tested in LBD.

Establishing whether DLB and PDD differ in white matter connections, and how these relate to plasma markers, has potential clinical implications, as it could determine whether particular patients would benefit from a common approach or more targeted interventions.

Our objective was to investigate whether white matter integrity and plasma biomarkers differ within LBD. We primarily aimed to test differences between DLB and PDD. In addition, we aimed to investigate whether measures differed based on presence of dementia in LBD and relative to healthy aging. We used fixel‐based analysis of diffusion‐weighted MRI, plasma NfL as a marker of axonal damage, and p‐tau217 as a marker of amyloid beta and tau co‐pathology. We compared each measure between (1) DLB and PDD, (2) LBD and PD with normal cognition who were characterized as low risk for developing PD dementia, and (3) LBD and age‐matched controls.

We predicted greater white matter integrity loss and increased concentrations of NfL and p‐tau217 in LBD compared to PD and age‐matched controls. Based on previous work using less‐sensitive FA measures, we predicted greater loss of white matter integrity and higher NfL in DLB than in PDD^12^; and based on increased amyloid beta pathology^23^ in DLB, we expected higher levels of p‐tau217 in DLB than PDD.

## 2 MATERIALS AND METHODS

### 2.1 Participants

People with DLB, PDD, and PD, and age‐matched controls 50–81 years of age were recruited from the National Hospital for Neurology and Neurosurgery outpatient clinics and affiliated hospitals, from national patient support groups (Lewy Body Society and Rare Dementia Support), and from an existing observational cohort study of PD (led by R.S.W.) (see e.g., Hannaway 2023^24^ for more details).

DLB and PDD participants had a clinical diagnosis according to Diamond Lewy toolkits for DLB or PDD.^25^ People with PD had a clinical diagnosis of PD according to the Movement Disorders Society (MDS) clinical diagnostic criteria.^26^ We further categorized people with PD into high and low risk for dementia, based on their performance on two computerized visual tasks: biological motion^27^ and the “Cats and Dogs task.”^28^ We have shown previously that poor performance on these tasks predicts dementia and poor outcomes in PD;^15^, ^24^ and that people with PD who are high risk for dementia already show extensive loss of white matter integrity and changes in plasma markers.^8^, ^15^ We therefore excluded people with PD categorized as high risk for dementia, based on performance on these tasks.

All patients were within 10 years of their respective diagnosis. For PDD, this was within 10 years of the dementia diagnosis. Where people with PD or controls had attended more than one visit in the longitudinal study, the last completed visit without dementia or mild cognitive impairment (MCI) was used. This was done to match ages to people with LBD. Where people with PD in the longitudinal study had developed PD‐MCI or dementia, their latest visit was used, and they were counted as PDD.

The following groups were included for analysis: PDD, *n* = 21); DLB, *n* = 47; LBD (*n* = 68), which is widely recognized as an umbrella term that includes both the PDD and DLB^1^; PD‐low risk for dementia: (*n* = 43) and controls (*n* = 21).

People with a history of confounding neurological or psychiatric disorders were excluded, as were metal implants considered unsafe for MRI. Controls diagnosed with dementia or MCI, or with a Mini‐Mental State Examination (MMSE) score of less than 25 were also excluded.

All participants provided written informed consent, and the study was approved by the Queen Square Research Ethics Committee (15.LO.0476).

### 2.2 Clinical and neuropsychological assessments

Participants underwent detailed clinical and neuropsychological assessments. The MMSE and Montreal Cognitive Assessment (MoCA) were completed as measures of global cognition, plus two tests per cognitive domain. These were: Attention: Stroop color naming^29^ and digit span^30^; Executive functions: category fluency^31^ and Stroop interference^29^; Language: letter fluency and graded naming task^32^; Memory: word recognition task^33^ and logical memory^30^; Visuospatial function: Hooper visual organization test^34^ and Benton Judgement of line orientation.^35^

**RESEARCH IN CONTEXT**

1. **Systematic review**: We reviewed the literature using PubMed. There is debate about whether dementia with Lewy bodies (DLB) and Parkinson's disease dementia (PDD) are separable diseases or a single Lewy body dementia (LBD) disease spectrum, but there are few studies directly comparing them. Fluid biomarkers and advanced magnetic resonance imaging (MRI) offer the potential to detect differences between these conditions.
2. **Interpretation**: We found MRI and plasma marker differences between LBD and cognitively intact Parkinson's, and also between DLB and PDD. Notably, MRI differences were distinct in the two comparisons. This suggests that DLB and PDD do differ in the underlying processes, and that these differences are distinct from the processes driving dementia in general in LBD.
3. **Future directions**: Larger studies comparing DLB and PDD, including measures that are sensitive to co‐pathologies, such as amyloid beta, tau, and small vessel disease, are likely to provide further insights into how DLB and PDD differ, with implications for future therapeutic interventions.

Due to higher frailty in people with LBD, it was not always possible to complete the full battery of neuropsychological testing during the study visit. Where this was the case, the MMSE and MoCA were prioritized, followed by one task per cognitive domain (in each case, the first of the two tests listed above was used). Furthermore, if participants were unable to complete the full Stroop task, they completed a “Half‐Stroop” consisting of the first three lines of the task.

**FIGURE 1:**
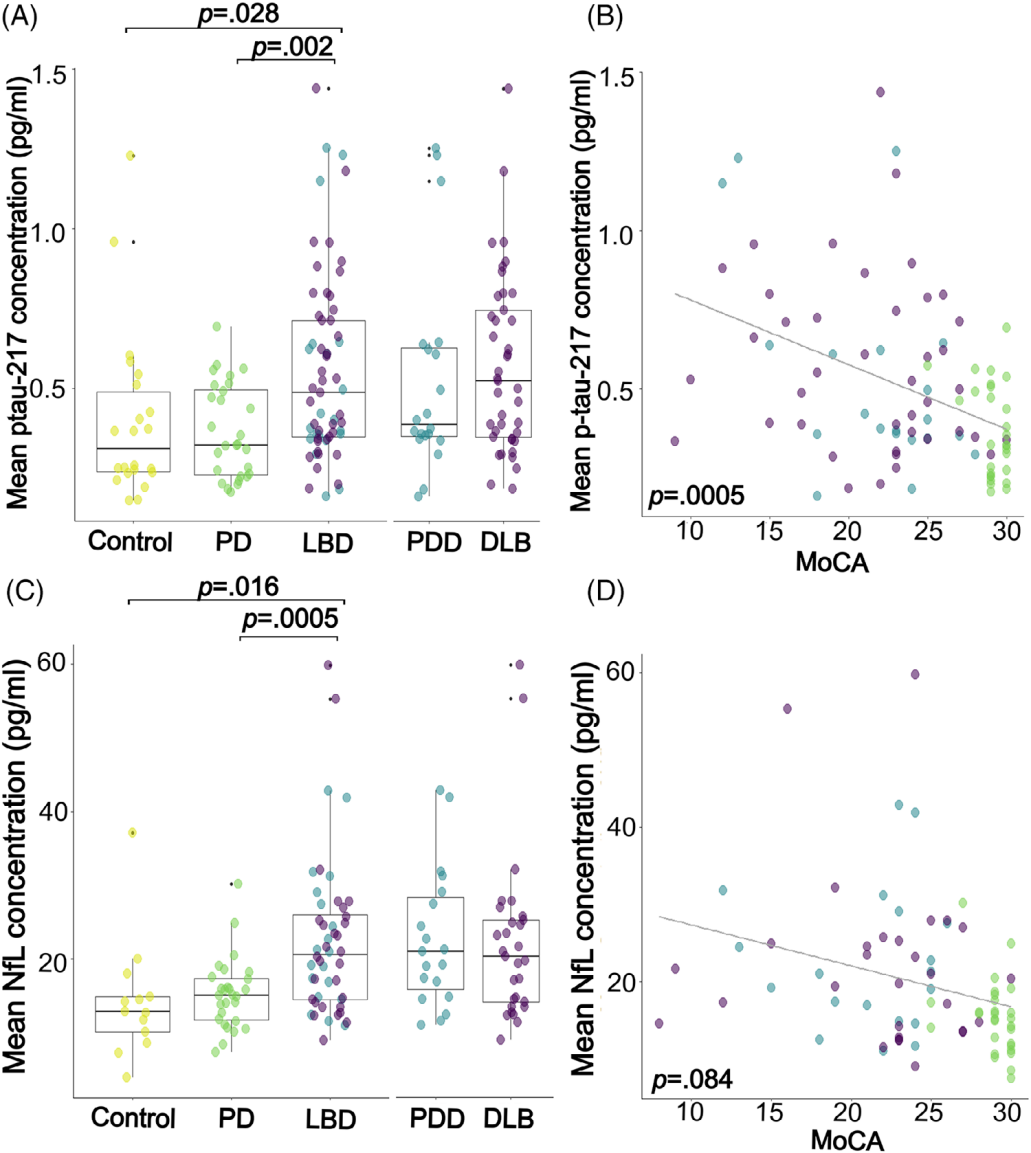
Plasma p‐tau217 and NfL in LBD. (A) Differences in plasma p‐tau217 concentrations between LBD, Parkinson's disease with low risk of dementia, and controls; and between LBD subtypes of PDD and DLB. (B) Relationship between plasma p‐tau217 and MoCA score in all patients (Purple = DLB, blue = PDD, green = PD‐low risk, as in A). (C) Differences in plasma NfL concentration between LBD, Parkinson's low risk and controls; and between LBD subtypes of PDD and DLB. (D) Relationship between NfL and MoCA score in all patients (Purple = DLB, blue = PDD, green = PD‐low risk, as in C). DLB, dementia with Lewy bodies, MoCA, Montreal Cognitive Assessment, NfL, neurofilament light; p‐tau217,  tau phosphorylated at threonine‐217; PD, Parkinson's disease, LBD, Lewy body dementia, PDD, Parkinson's disease dementia.

A summary cognitive score was calculated as the averaged *z*‐scores of the MoCA plus one task per cognitive domain: namely inverted Stroop (color naming time), category fluency, letter fluency, Recognition Memory Test, and Hooper Visual Organization Test, as we have described previously.^24^ Where only Half‐Stroop was completed, time to complete the full‐Stroop was predicted using a regression model to allow calculation of the summary cognitive score (further details in the Supplementary Methods, Figure 1).

Motor symptom severity was measured using the United Parkinson's Disease Rating Scale, part 3 (UPDRS‐III),^36^ and the timed up and go test (TUG).^37^ Total symptoms were measured using the UPDRS parts I, II, III, and IV. For all clinical and cognitive measures, participants were tested while taking their usual medications, and Levodopa Equivalent Daily Dose (LEDD) was calculated.^38^

Cognitive fluctuations were measured using the clinician assessment of fluctuations (CAF), one‐day fluctuations scale,^39^ and the dementia cognitive fluctuation scale (DCFS).^40^ Anxiety and depression were measured using the hospital anxiety and depression scale (HADS).^41^ Impairments in activities of daily living were measured using the Functional Activities Questionnaire^42^; autonomic symptoms using Compass‐31^43^; sleep disturbances using the Rapid Eye Movement Behavior Disorder Sleep Questionnaire (RBDSQ)^44^; and visual hallucinations using the University of Miami PD Hallucinations Questionnaire (UMPDHQ).^45^

Best corrected visual acuity, wearing habitual lenses if worn, was assessed using the LogMAR chart at 3 m viewing distance. Contrast sensitivity was measured using the Pelli–Robson chart (SSV‐281‐PC) (http://www.sussex‐vision.co.uk) at 1 m viewing distance. Color vision was tested using the Farnsworth D15 test.^46^

### 2.3 Plasma collection and processing

Approximately 30 mL of blood was collected in polypropylene EDTA tubes. Samples were centrifuged at 2000 g for 10 min, generating up to 16 mL × 0.5 mL plasma and 14 mL × 0.5 mL serum aliquots, and stored immediately at −80°C.

NfL concentrations were measured using the Simoa Human Neurology 4‐Plex A (N4PA) assay (Quanterix). Eighty‐nine participants had a sample available for inclusion in NfL analysis (30 people with DLB, 19 PDD, 27 PD‐low risk, and 13 controls). Concentration of p‐tau217 was measured using the AlzPath Simoa HD‐X *p‐*Tau‐217 Advantage‐PLUS kit.^47^ One hundred eleven participants had a sample available for inclusion in p‐tau217 analysis (42 DLB, 20 PDD, 27 PD‐low risk, and 22 controls). All measurements were performed by analysts blinded to participant’ diagnoses and clinical data. Measurements of p‐tau217 were performed in a single batch of reagents and NfL (4‐Plex) measurements were performed in four batches of reagents.

### 2.4 MRI scanning

All MRI data were acquired using the same 3T Siemens Magnetom Prisma scanner with a 64 channel receive array coil (Siemens Healthcare, Erlangen, Germany). Participants underwent ∼1 h of scanning while receiving their usual medication. Structural anatomic scans consisted of T1‐weighted magnetisation‐prepared rapid gradient‐echo (MPRAGE) images, acquired using 1 mm × 1 mm × 1 mm voxel, matrix dimensions 256 × 256 × 176, echo time (TE) = 3.34 ms, repetition time (TR) = 2530 ms, flip angle = 7 degrees. DWI was acquired using 2 × 2 × 2 mm isotropic voxels, matrix dimensions 220 × 220 × 72, TE = 3260 ms, TR = 58 ms, *b* = 50 s/mm^2^/17 directions, *b* = 300 s/mm^2^/8 directions, *b* = 1000 s/mm^2^/64 directions, b = 2000 s/mm^2^/64 directions, and acceleration factor = 2.

### 2.5 Image analysis

#### 2.5.1 Preprocessing

Diffusion MRI images were pre‐processed using Mrtrix3 by denoising,^48^ removal of Gibbs ringing artefacts,^49^ eddy current correction, motion correction,^50^ and bias‐field correction.^51^ Diffusion‐weighted images were up‐sampled to a spatial resolution of 1.3 mm^3^.

#### 2.5.2 Fixel‐based analysis of diffusion‐weighted imaging data

Fiber orientation distributions (FODs) were computed using multi‐shell three‐tissue‐constrained spherical deconvolution using the group‐average response function for each tissue type (gray matter, white matter, and cerebrospinal fluid).^52^, ^53^ Each participant's FOD image was registered to a study‐specific template using affine and then nonlinear registration, and a template mask image calculated as the intersection of the brain masks for each participant.

Whole brain probabilistic tractography was performed on the FOD template to generate a tractogram with 20 million streamlines. To reduce tractography biases, this was then filtered to 2 million streamlines using spherical‐deconvolution informed filtering of tractograms (SIFTs).^54^

Fixel‐based analysis was performed to characterise white matter tracts. For each participant, three measures were derived:

1. Fiber cross‐section (FC): a measure of macrostructure.^55^
2. Fiber density (FD): a measure of microstructural changes within tracts.^55^
3. Fiber density and cross‐section (FDC): a combined measure of change at both the micro‐ and macro‐structural levels.^55^

Fixel‐derived metrics were compared between people with (1) DLB and PDD, (2) LBD and PD‐low risk, and (3) LBD and controls, using age, sex, and total intracranial volume as covariates (although not for comparisons involving FD, as recommended).^56^, ^57^ We also performed correlational analyses across all people with LBD and PD‐low risk, with motor and cognitive scores. To check that correlational results were not driven by group differences, correlational analyses were repeated using the LBD group only. All results are reported as *p*_FWE_ < 0.05.

#### 2.5.3 Voxel‐based analysis

A voxel‐based analysis of fractional anisotropy (FA) and mean diffusivity (MD) was also performed. The diffusion tensor was calculated from the preprocessed diffusion images and used to derive FA and MD for each subject. The maps of each subject were registered to a study‐specific template and voxel‐wise analysis was conducted. Voxel‐derived analysis was performed for the same comparisons and covariates as the fixel‐derived analysis, using threshold‐free cluster enhancement with default parameters.^58^ All results are reported as *p*_FWE_ < 0.05.

### 2.6 Other statistical analyses

#### 2.6.1 Demographics and plasma markers

Demographic, cognitive, and clinical measures were compared between groups using two‐tailed Welch's *t*‐tests or Mann–Whitney tests for non‐normally distributed data.

Plasma data were matched to clinical phenotype data, and a comparison made between disease groups, using one‐way analysis of variance (ANOVA), controlling for age and sex. Planned comparisons were conducted to compare (1) DLB versus PDD, (2) LBD versus PD‐low risk, and (3) LBD versus controls. Associations between plasma measures and cognitive/clinical scores were tested using linear regression, adjusted for age and sex. *P* < 0.05, Bonferroni‐corrected for multiple comparisons (*p* < 0.025 for group comparisons) was accepted as the threshold for statistical significance. Analyses were performed in *R* (R‐4.2.1; https://www.r‐project.org/).

## 3 RESULTS

### 3.1 Demographics and clinical severity

A total of 47 DLB, 21 PDD, 43 PD, and 23 controls were recruited. Fourteen PD were classified as high risk for developing dementia and were excluded, as they have been shown to have diffuse changes in white matter connections.^8^, ^15^ This left 29 PD‐low risk.

The ages of people with DLB, PDD, and controls did not differ significantly (mean ± SD, DLB: 72.1 ± 5.8, PDD: 72.1 ± 6.4, control: 72.8 ± 6.8; DLB/PDD: *t* = 0.20, *p* = 0.84; LBD/controls: *t* = 0.585, *p* = 0.56), but PD‐low risk (67.6 ± 5.1) were younger than people with DLB and PDD, *t* = 3.59, *p* = 0.0006. There was a higher proportion of men with DLB and PDD than either PD‐low risk (*χ* ^2^ = 33.69, *p* < 0.0001) or controls (*χ* ^2^ = 13.8, *p* = 0.0002). MoCA scores did not differ between people with DLB (21.3 ± 5.3) and PDD (21.8 ± 4.4) (*W* = 464.5, *p* = 0.80). As expected, MoCA scores were reduced for people with LBD compared to people with PD‐low risk (28.9 ± 1.4; W = 74, *p* < 0.0001) and controls (28.9 ± 1.1; *W* = 1492.5 *p* < 0.0001). Motor severity as measured using UPDRS‐III was greater in people with LBD (PDD: 38.9 ± 1.11, DLB: 34.1 ± 17.6) than PD‐low risk (26.0 ± 10.4; *W* = 1338, *p* = 0.009) and control (4.2 ± 3.5; *W* = 20, *p* < 0.0001) groups. Demographics and clinical assessments are shown in Table 1. Cognitive measures and clinical questionnaires are shown in Table 2.

**TABLE 1:** Demographic and clinical information of participants.

	PDD (*n* = 21)	DLB (*n* = 47)	LBD (*n* = 68)	PD‐low risk (*n* = 29)	Controls (*n* = 23)	PDD/DLB	LBD/PD	LBD/Control
Age	72.1 (6.43)	72.1 (5.75)	72.1 (5.91)	67.6 (5.06)	72.8 (6.78)	0.85	**<0.0001**	0.61
Gender	17 M/4 F	43 M/4 F	60 M/8 F	9 M/20 F	11 M/12 F	0.40	**<0.0001**	**0.0001**
Years of education	15.4 (3.58)	15.0 (3.25)	15.1 (3.33)	16.2 (3.38)	16.0 (2.31)	0.73	0.17	0.32
Disease‐specific metrics								
UPDRS‐III	38.9 (11.1)	34.1 (17.6)	35.6 (15.9)	26.0 (10.4)	4.18 (3.51)	0.14	**0.003**	**<0.0001**
UPDRS‐total	80.2 (22.7)	66.1 (30.1)	70.4 (28.6)	50.3 (18.9)	6.48 (4.23)	**0.024**	**0.0008**	**<0.0001**
LEDD	705.2 (387.2)	273.8(261.0)	406.6 (362.7)	597.1 (343.2)	0 (0)	**<0.0001**	**0.008**	**<0.0001**
Disease duration parkinsonism	8.38 (5.57)	2.15 (2.03)	4.10 (4.55)	7.04 (3.13)	–	**<0.0001**	**<0.0001**	–
Disease duration dementia	1.67 (1.98)	2.17 (2.05)	2.02 (2.03)	–	–	0.022	–	–
Global cognitive measures								
MMSE	26.5 (3.20)	25.2 (3.88)	25.6 (3.71)	28.3 (5.52)	29.2 (1.03)	0.10	**<0.0001**	**<0.0001**
MoCA	21.8 (4.41)	21.3 (5.34)	21.5 (5.04)	28.9 (1.37)	28.9 (1.10)	0.92	**<0.0001**	**<0.0001**
Composite Cognitive Score	−2.09 (1.57)	−2.77 (1.75)	−2.56 (1.71)	0.20 (0.64)	0.07 (0.47)	0.15	**<0.0001**	**<0.0001**

*Note*: All values are mean (standard deviation), apart from gender, which is shown as a proportion. LBD = Lewy body dementia (consisting of PDD and DLB. Note that these are the same patients as described in the separated DLB and PDD columns). For people with PDD, disease duration is specified separately for onset of motor Parkinsonism and onset of dementia. Significant differences between groups are in bold.

Abbreviations: DLB, dementia with Lewy bodies; LBD, Lewy body dementia; MMSE, Mini‐Mental State Examination, MoCA, Montreal Cognitive Assessment, LEDD, levodopa equivalent daily dose, PD‐low risk, Parkinson's disease, at low risk of developing dementia; PDD, Parkinson's disease dementia; UPDRS‐III, Unified Parkinson's Disease Rating Scale Part 3 (motor assessment); UPDRS‐total, Unified Parkinson's Disease Rating Scale total symptom score.

**TABLE 2:** Cognitive and questionnaire scores.

	PDD (*n* = 21)	DLB (*n* = 47)	LBD (*n* = 68)	PD‐low risk (*n* = 29)	Controls (*n* = 23)	PDD/DLB	LBD/PD	LBD/Control
Cognitive measures								
Digit span backwards	5.7 (2.0)	5.5 (2.6)	5.5 (2.4)	8.2 (3.0)	7.6 (2.3)	0.59	**0.0001**	**0.003**
(Hal Stroop color naming	27.3 (9.2)	28.2 (10.3)	27.9 (10.0)	17.5 (3.1)	17.4 (2.5)	0.62	**<0.0001**	**<0.0001**
Stroop interference	69.5 (48.5)	71.1 (48.6)	70.0 (48.1)	31.5 (9.6)	32.2 (5.4)	0.78	**<0.0001**	**<0.0001**
Verbal fluency category	12.3 (4.8)	10.3 (4.8)	10.9 (4.8)	22.3 (4.7)	21.1 (5.4)	0.11	**<0.0001**	**<0.0001**
Graded naming task	19.1 (5.5)	21.0 (5.8)	20.4 (5.7)	25.1 (3.0)	26.0 (3.2)	0.12	**<0.0001**	**<0.0001**
Verbal fluency letter	13.0 (4.8)	10.4 (5.4)	11.2 (5.3)	18.8 (4.7)	17.7 (5.7)	0.06	**<0.0001**	**<0.0001**
Word recognition task	21.6 (5.6)	21.9 (3.0)	21.8 (4.0)	24.6 (0.9)	24.6 (0.8)	0.50	**<0.0001**	**<0.0001**
Logical memory (delayed)	8.4 (4.3)	7.0 (4.6)	7.2 (4.5)	15.1 (4.5)	15.9 (4.0)	0.36	**<0.0001**	**<0.0001**
Hooper visual Organization	13.7 (7.1)	17.7 (6.0)	16.5 (6.5)	26.2 (3.3)	25.1 (2.4)	**0.031**	**<0.0001**	**<0.0001**
Clinical questionnaires								
HADS depression Score	6.8 (4.5)	5.8 (3.2)	6.1 (3.6)	3.8 (2.8)	2.8 (3.0)	0.51	**0.003**	**<0.0001**
HADS anxiety score	8.2 (5.6)	5.5 (3.9)	6.2 (4.5)	5.2 (3.4)	4.4 (3.9)	0.09	0.43	0.09
Hallucinations (UMPDHQ)	2.2 (3.3)	3.9 (3.0)	3.4 (3.2)	0.9 (2.5)	0 (0)	**0.019**	**<0.0001**	**<0.0001**
Sleep (RBDSQ)	6.8 (3.9)	7.4 (3.5)	7.2 (3.6)	4.1 (2.9)	1.6 (1.5)	0.45	**0.0002**	**<0.0001**
Fluctuations (CAF)	3.3 (3.2)	5.9 (4.4)	5.3 (4.3)	–	–	0.09	–	–
Functional activities	11.6 (7.4)	10.8 (6.6)	11.0 (2.1)	2.1 (3.0)	0.2 (0.5)	0.65	**<0.0001**	**<0.0001**

*Note*: All values are mean (standard deviation). (Consisting of PDD and DLB. Note that these are the same patients as described in the separated DLB and PDD columns.) Significant differences between groups are in bold.

Abbreviations: CAF, clinician assessment of fluctuations; Combined LBD, Lewy body dementia; DLB, dementia with Lewy bodies; HADS, Hospital Anxiety and Depression Scale; PD‐low risk, Parkinson's disease, at low risk of developing dementia; PDD, Parkinson's disease dementia; RBDSQ, REM Sleep Behavior Disorder Questionnaire.

### 3.2 Plasma markers in LBD

#### p‐tau217

Forty‐two DLB, 20 PDD, 27 PD‐low risk, and 22 control participants had a sample available for inclusion in the plasma p‐tau217 analysis. People with LBD showed higher concentrations of plasma p‐tau217 than people with PD‐low risk (*F *= 10.80, *p *= 0.002) and age‐matched controls (*F *= 5.04, *p *= 0.028) (Figure 1A). Concentrations of p‐tau217 did not differ between people with DLB and PDD (*F *= 0.15, *p *= 0.70). Across all patient groups, plasma p‐tau217 was significantly associated with cognition, corrected for age and sex (MMSE: β = −4.62, *p* = 0.012; MoCA: β = −7.06, *p* = 0.0005; cognitive composite: β = −2.47, *p* = 0.002; Figures 1B, S2A and S2B), but was not associated with motor or total symptom scores (UPDRS‐total: β = 8.21, *p* = 0.75; UPDRS‐III: β = 1.65, *p* = 0.42). This pattern of results remained the same when associations were tested in the LBD group only (Supplementary Results; Figure S3A).

#### NfL

Thirty people with DLB, 19 PDD, and 27 PD‐low risk, and 13 control participants had a sample available for inclusion in the plasma NfL analysis. There was a significant effect of age on plasma NfL concentration (β = 0.47, *p* = 0.011). There was no effect of reagent batch or sex. Mean concentration of plasma NfL was significantly greater in LBD than in PD‐low risk (*F *= 13.26, *p *= 0.0005, Figure 1C) and controls (*F *= 6.36, *p *= 0.016), when controlling for age and sex. Plasma NfL concentration did not differ significantly between people with DLB and PDD (*F *= 2.56, *p *= 0.12). Across all patient groups, plasma NfL concentration was significantly associated with the cognitive composite score (β = −0.06, *p* = 0.024, Figure S1D), but not with any other clinical scores, corrected for age and sex (MMSE: β = −0.03, *p* = 0.66; MOCA: β = −0.11, *p* = 0.08; UPDRS‐total: β = 0.33, *p* = 0.35; UPDRS‐III: β = 0.15, *p* = 0.41; Figures 1D and S2C). This pattern of results remained the same when associations were tested in the LBD group only (Supplementary Results; Figure S3B).

#### 3.2.1 White matter connections in LBD

##### 3.2.1.1 Fixel‐based analysis

##### LBD versus PD‐low risk

**FIGURE 2:**
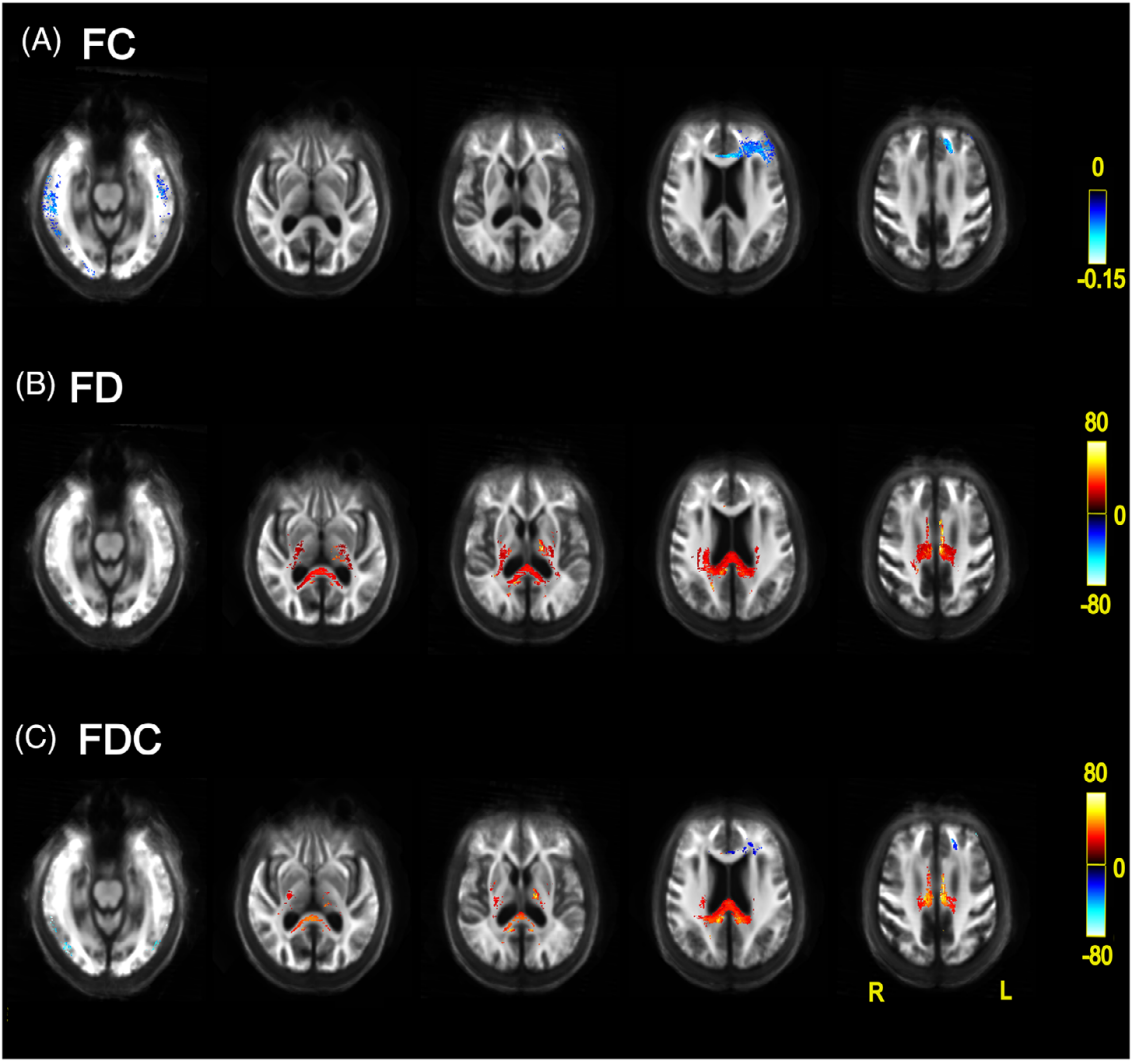
Differences in fixel‐based metrics between people with PD and LBD. Percentage change for LBD compared with PD in (A) FD, (B) FC, and (C) combined FDC. Red–yellow colors indicate *increases* in fixel‐based metrics and blue colors indicate *decreases* for LBD relative to the PD group. All results are displayed as streamlines corresponding to fixels that differed significantly between LBD and PD‐low risk groups (*p*_FWE_ < 0.05). Streamlines are displayed on the group white matter template and colored by percentage change (color bars). FC, fiber cross‐section; FD, fiber density; FDC, fiber density and cross‐section; FWE, family‐wise error corrected; LBD, Lewy body dementia; PD, Parkinson's disease.

People with LBD showed reduced FC compared to PD‐low risk in the genu of the corpus callosum and left anterior corona radiata, and bilaterally in the sagittal stratum and posterior thalamic radiation (Figure 2A). No regions showed increased FC for LBD groups.

FD was reduced for LBD relative to PD‐low risk in the temporal and occipital regions. FD was greater in the LBD group relative to PD‐low risk in the genu, body, and splenium of the corpus callosum, bilateral posterior limb, and retro‐lenticular part of internal capsule, bilateral posterior and superior corona radiata, cingulum, right external capsule, right superior longitudinal fasciculus, and bilateral cerebral peduncle (Figure 2B).

##### PDD versus DLB

**FIGURE 3:**
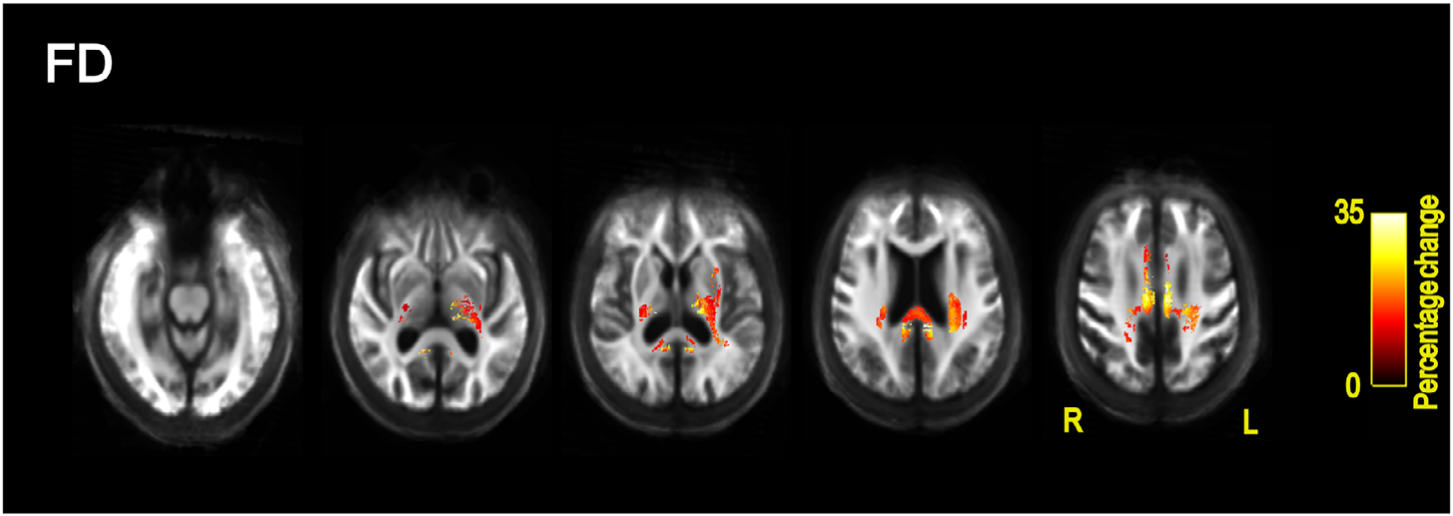
Fixel‐based reductions in fiber density for PDD relative to DLB. All results are displayed as streamlines corresponding to fixels that showed significantly reduced fiber density for the PDD relative to DLB group (*p*_FWE_ < 0.05). Streamlines are displayed on the group white matter template and colored by percentage change (color bars). DLB, dementia with Lewy bodies; FWE,  family‐wise error corrected; PDD, Parkinson's disease dementia.

People with PDD showed reduced FD compared to DLB in the body and splenium of the corpus callosum, cingulum, bilateral posterior limb and retrolenticular part of internal capsule, bilateral superior and posterior corona radiata, posterior thalamic radiation, left superior longitudinal fasciculus, left external capsule, and left cerebral peduncle (Figure 3).

There were no regions that showed reduced FD for people with DLB relative to PDD. No differences were observed in FC between the two groups.

##### LBD versus controls

No differences in fixel‐based metrics were observed between the LBD and control groups.

##### Association with plasma concentrations and clinical variables

**FIGURE 4:**
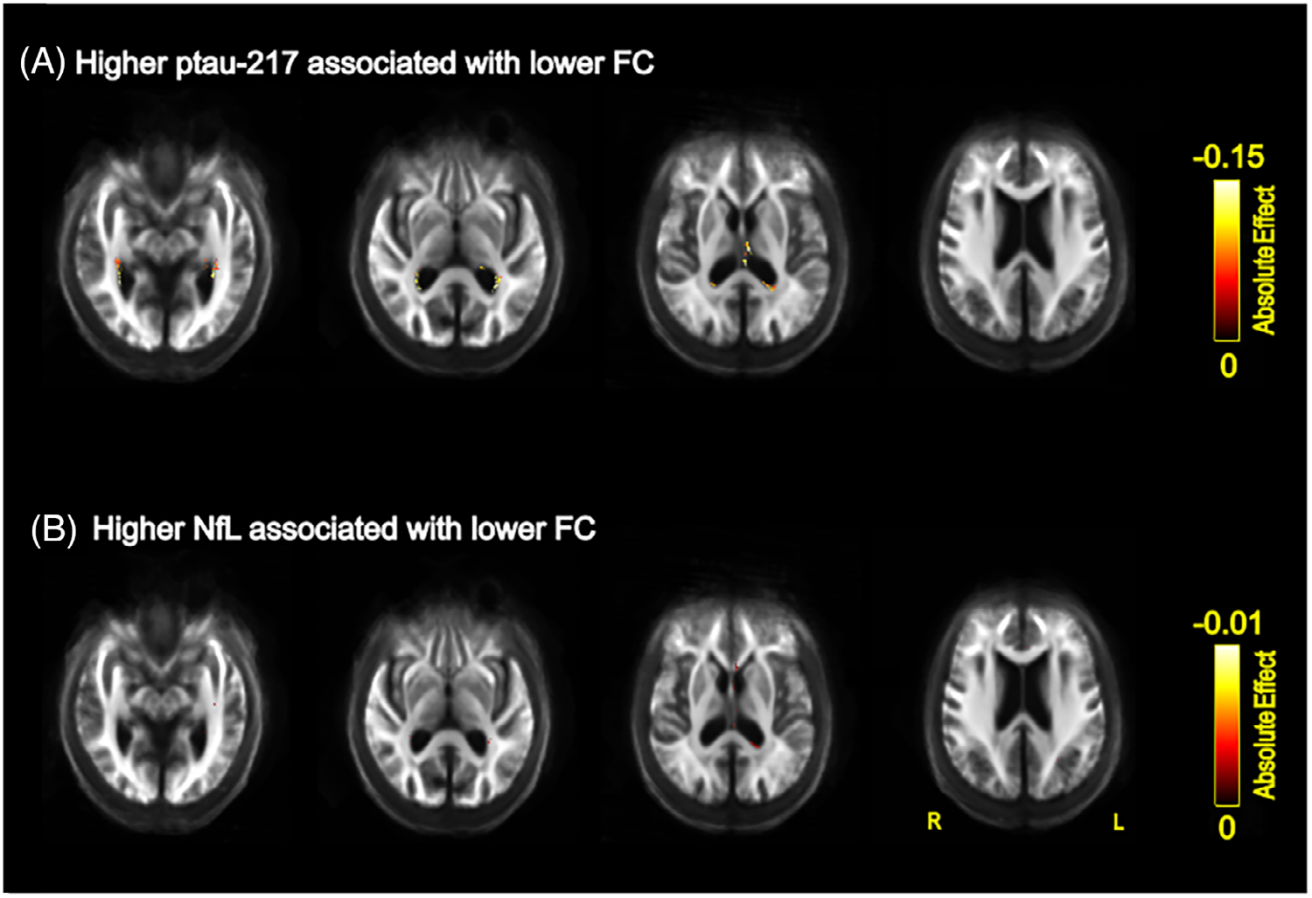
Associations between fixel‐based FC and plasma measurements. Association of (A) increased concentrations of plasma p‐tau217 with reduced fiber cross‐section, (B) increased concentrations of plasma NfL chain with reduced fiber cross‐section. All results are displayed as streamlines corresponding to fixels that were significantly associated with plasma measures (*p*_FWE _< 0.05). Streamlines are displayed on the group white matter template and colored by absolute effect (color bars). FC, fiber cross‐section; NfL, neurofilament light; FWE, family‐wise error corrected.

Higher concentrations of plasma p‐tau217 were associated with reduced FC in people with LBD and PD‐low risk in the splenium of the corpus callosum, fornix and bilaterally with the retro‐lenticular part of internal capsule (Figure 4), posterior thalamic radiation, sagittal stratum, and tapetum. Likewise, higher concentrations of plasma NfL were associated with reduced FC in the genu and splenium of the corpus callosum, posterior thalamic radiation, sagittal stratum, and the right retro‐lenticular part of internal capsule. Neither plasma measure was associated with FD in people with LBD and PD. In addition, higher concentrations of plasma NfL and p‐tau217 were associated with lower FC when associations were tested in the LBD group only (Supplementary Results; Figure S4).

**FIGURE 5:**
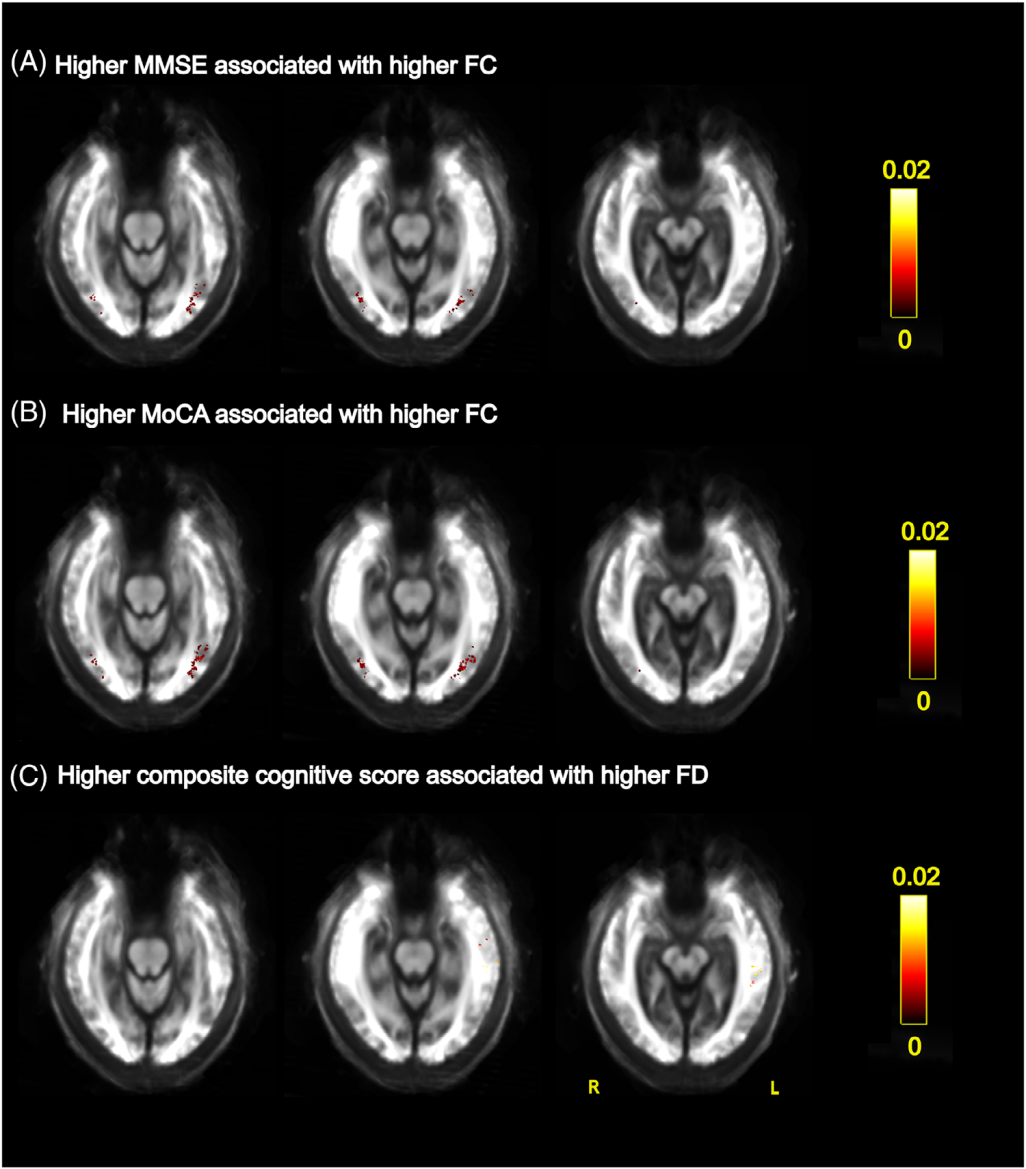
Associations between fixel‐based fiber cross‐section (FC) and cognitive measurements. Association of (A) higher MMSE score with higher FC, (B) higher MoCA score with higher FC, and (C) higher composite cognitive score with higher FC. All results are displayed as streamlines corresponding to fixels that were significantly associated with cognitive measures (*p*_FWE _< 0.05). Streamlines are displayed on the group white matter template and colored by absolute effect (color bars). FC, fiber cross‐section; FWE, family‐wise error corrected; MoCA, Montreal Cognitive Assessment; MMSE, Mini‐Mental State Examination.

Significant associations were observed between FC and cognition in people with LBD and PD, although these were found in both directions (Figure 5): lower FC, indicating reduced white matter macrostructure, was associated with lower MMSE and MoCA bilaterally in the posterior thalamic radiation and with lower cognitive composite scores in the left sagittal striatum. In addition, higher FD, indicative of preserved microstructure, was associated with poorer MoCA score, but also with higher cognitive composite score in the left superior longitudinal fasciculus (Figure S5). Higher UPDRS‐III scores, reflecting worse motor symptoms, were associated with lower FC in the cingulum (Figure S6). No association was seen between UPDRS‐Total scores and FC or FD measures. Associations of clinical measures with fixel‐based metrics are presented in the Supplemental Results and Figures S7 and S8.

#### 3.2.2 Voxel‐based analysis

**FIGURE 6:**
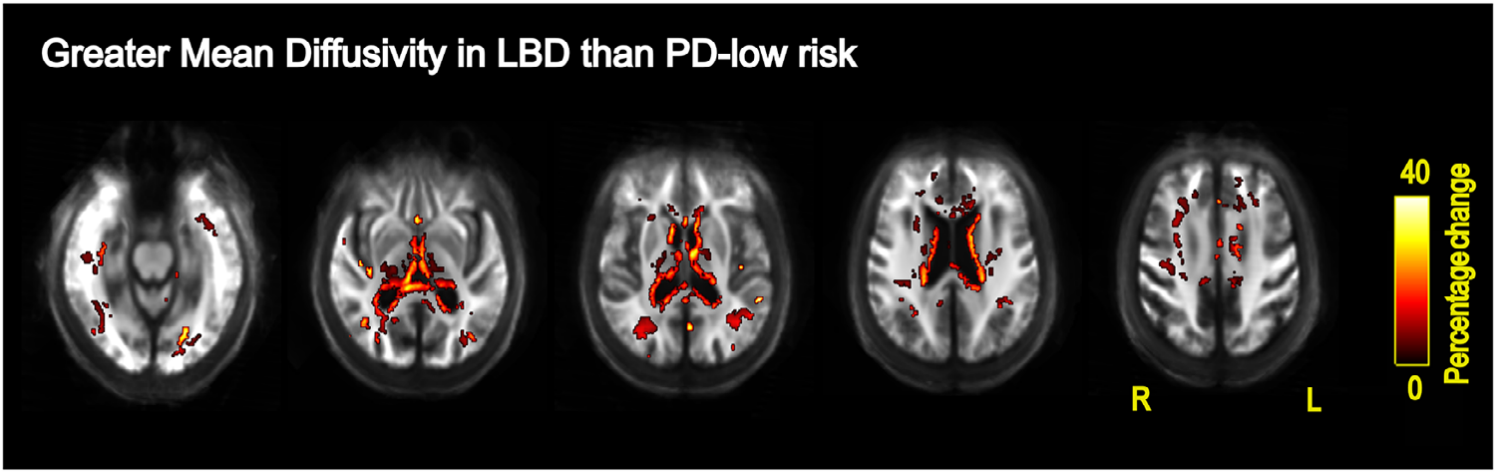
Voxel‐based increases in mean diffusivity for LBD relative to PD with low dementia risk. All results are displayed as voxels that showed significantly increased mean diffusivity for the LBD relative to PD‐low risk group (*p*_FWE _< 0.05). Voxels are displayed on the group white matter template and colored by percentage change (color bars). FWE, family‐wise error corrected; LBD, Lewy body dementia; PD, Parkinson's disease.

Conventional voxel‐based analysis did not show any statistically significant differences between people with DLB and PDD after FWE correction. People with LBD showed increased mean diffusivity (MD) relative to people with PD‐low risk, in widespread areas including the body and genu of corpus callosum, fornix, tapetum, superior fronto‐occipital fasciculus bilaterally within the anterior, superior and posterior corona radiata, anterior and posterior limb of internal capsule, posterior thalamic radiation, superior longitudinal fasciculus, sagittal stratum, and in the right cerebral peduncle (Figure 6). No differences in FA were observed between the LBD and PD‐low risk groups. No statistically significant differences in FA or MD were observed between LBD and controls after FWE correction.

## 4 DISCUSSION

We examined differences in diffusion imaging and plasma markers between people with LBD subtypes of DLB and PDD; and between people with LBD and people with PD who were low risk for dementia. We show macrostructural (fiber cross‐section) changes, increased plasma NfL, and increased plasma *p*‐tau217 in people with LBD relative to PD‐low risk. We also found bidirectional microstructural changes (fiber density) in people with LBD relative to PD‐low risk. When comparing LBD subtypes, we found greater microstructural changes (FD) in PDD relative to DLB. There were no differences in plasma measures, or macrostructure (FC) between LBD subtypes. These findings suggest a distinction between reduced white matter macrostructure in the presence of dementia in LBD versus microstructural differences between DLB and PDD groups.

Our findings of reduced fiber cross‐section in LBD relative to PD‐low risk is consistent with our previous work: we used higher‐order diffusion models and showed reduced fiber cross‐section in PD‐MCI and in PD at greater dementia risk,^15^ compared to PD low‐risk for dementia. Other groups used less‐sensitive diffusion tensor imaging (DTI) approaches to compare LBD and PD and showed reduced FA in the corpus callosum, longitudinal fasciculus, and fronto‐occipital fasciculus for PDD and PD‐MCI compared to PD with intact cognition.^10^, ^11^ However, there are no other studies that apply whole‐brain comparisons of the more sensitive higher‐order DWI techniques. Similarly, DLB has not been investigated previously using fixel‐based analysis.

Reductions in fiber cross section are thought to represent axonal loss, and reflect changes in the area perpendicular to white matter bundles.^14^ These changes have been linked with neurodegeneration and seem to be related to amyloid status.^59^ Consistent with this observation, we found reductions in fiber cross‐section for LBD compared to PD low risk for dementia, where previous research shows greater neurodegeneration, measured using gray matter atrophy,^60^ and higher rates of amyloid positivity than in PD.^61^ This is also supported in our work by higher plasma p‐tau217 and NfL in LBD compared with PD‐low risk for dementia.

Our plasma p‐tau217 findings are noteworthy, as they represent, to our knowledge, the first report of plasma p‐tau217 concentrations in LBD and PD. Our findings are consistent with previous research using earlier versions of plasma p‐tau (thought to be less sensitive). These showed increased p‐tau181 relative to controls in DLB^22^ and PDD,^19^ and higher p‐tau181 concentrations associated with cognition in DLB.^22^

We also showed increased plasma NfL in LBD, consistent with previous work showing that NfL is increased in DLB^18^ and PD with cognitive impairment.^19^, ^62^ NfL is a marker of axonal damage,^17^ and we show that it is associated with changes in white matter macrostructure (FC). Of interest, we did not find an association between NfL and MMSE or MoCA, which has been shown in some previous work ^19^ in LBD, although we did find an association of NfL with a composite cognitive score, which is more sensitive to subtle cognitive deficits.

Our finding of both increased and decreased fiber density for LBD relative to PD at low risk of dementia was unexpected, with increased fiber density in regions including the corpus callosum and internal and external capsule, and decreases in temporal and occipital tracts. Some authors have suggested a role for compensatory white matter alterations in PD.^63^, ^64^ Another explanation relates to differences in small vessel disease (SVD) between PD‐low risk and LBD groups. Fiber density is thought to reflect intra‐axonal volume changes.^14^ In a recent Alzheimer's study, microstructural differences were associated with SVD, with fiber density strongly associated with white matter hyperintensities, lacunas, and cerebral microbleeds across several tracts.^59^ SVD changes may differentially affect outcomes depending on their location. In the present study, reduced FD was seen in the temporal and occipital regions. In these same regions, greater white matter hyperintensity burden has been shown to be associated with poorer cognitive function in older adults,^65^ and with amyloid positivity in MCI.^66^ Likewise, in PD, enlarged perivascular spaces in temporal regions related to cognitive scores, whereas those in the centrum semiovale did not.^67^ In our cohort, we did not have imaging sequences available that were sensitive to SVD, and this could be examined in future work.

When we compared DLB and PDD, we found reductions in fiber density in PDD relative to DLB, indicating microstructural differences. Fiber density differences were observed in corpus callosum, corona radiata, internal capsule, and superior longitudinal fasciculus, regions where DTI white matter changes were observed previously in PDD and PD‐MCI compared to PD and controls.^10^, ^11^ This suggests that in our cohort, differences between DLB and PDD were not associated with amyloid status or neurodegeneration (linked with *macrostructure*), but there may instead be differences in microstructure, associated with SVD.

We found widespread changes in mean diffusivity in LBD relative to PD at low risk for dementia, consistent with previous findings of changes in DTI metrics in both DLB^9^, ^11^, ^12^ and PDD.^10^, ^11^, ^12^ However, DTI models lack specificity, as they are unable to model crossing fibers. Observed increases in mean diffusivity could be caused by multiple different processes, such as demyelination, axonal loss, or inflammation.^68^ Our fixel‐based analysis instead allows us to interpret the observed MD changes with greater specificity and show that both macro‐ and microstructural changes are relevant white matter differences observed between LBD and PD with low dementia risk.

## 5 PRACTICAL IMPLICATIONS AND SIGNIFICANCE OF THIS WORK

The question of whether DLB and PDD are the same disease or two separate conditions continues. Our work now shows that differences between DLB and PDD can be measured in vivo, and that SVD may be a relevant factor. By better understanding disease processes in LBD, our research could have implications for future clinical practice, with potential for more tailored treatment approaches in LBD.

### 5.1 Limitations and future directions

There are some potential limitations to consider within this study. We were unable to quantify concurrent SVD, which could help understand changes that we observed in fiber density. Future research should include fluid‐attenuated inversion recovery or T2 MRI sequences to address this.

Our control and PD with low risk groups differed in age and sex to LBD groups. Although we corrected for these factors in all analyses, it would have been preferable to recruit matched groups. All image acquisition was completed with participants taking their usual dopaminergic medication, in order not to affect cognitive scores. However, all comparisons are based on structural imaging measures, which are unlikely to be affected by dopaminergic agents.

### 5.2 Conclusion

In summary, we show reduced white matter macrostructure and increased plasma p‐tau217 and NfL in LBD compared with PD with low risk for dementia, and both of these measures were associated with poorer cognition. We also show poorer white matter microstructure for PDD relative to DLB. Our findings suggest both micro‐ and macro‐structural changes in LBD. The observed microstructural changes suggest that SVD may differ between LBD sub‐types. Future work that is sensitive to SVD may help to further clarify these differences.

## CONFLICTS OF INTEREST STATEMENT

R.S.W. has received speaking and writing honoraria from G.E. Healthcare, Bial, Omnix Pharma, and Britannia; and consultancy fees from Therakind and Accenture. All other authors report no competing interests. Author disclosures are available in the Supporting Information.

## CONSENT STATEMENT

All participants provided written informed consent, and the study was approved by the Queen Square Research Ethics Committee (15.LO.0476).

## Supporting information



Supporting Information

Supporting Information
