# Supplementary material for: Neuroimaging and plasma biomarker differences and commonalities in Lewy body dementia subtypes
Source: Alzheimers Dement. 2025 May 22;21(5):e70274. doi: 10.1002/alz.70274 (PMC12097529; doi:10.1002/alz.70274)

# **Supplementary Materials**

## **Supplementary Methods**

If participants were unable to complete the full Stroop task, they completed a “Half-Stroop” consisting of the first 3 lines of the task. For participants who only completed a Half-Stroop, time to complete the full-Stroop colour naming was predicted using a regression model, derived from the full set of participant data, incorporated Half-Stroop time, age, sex and diagnosis. Missing full-Stroop times were predicted by combining the coefficients from this model with time to complete half-Stroop, age, sex and diagnosis. Predicted Stroop time was highly correlated with actual Stroop time in participants for which both measures were available (R^2^=0.97, Supplementary Figure 1), and predicted values were very close to actual values (RMSE = 4.65).

Additionally, if participants were unable to complete one task needed for the composite cognitive score, this was substituted with the mean z-score of that task for their disease group. If more than one task was not completed, the composite cognitive score was not calculated for that individual.

## **Supplementary Results**

### **Plasma analysis**

Within the LBD group, plasma p-tau217 was significantly associated with MMSE (β=-3.43, p=.043) and MOCA score (β=-4.56, p=.042), but was not associated with cognitive composite score (β=-0.91, p=.26), controlling for age and sex. Plasma p-tau217 was not associated with motor or total symptom scores (UPDRS-total: β=0.86, p=.94; UPDRS-III: β=0.76, p=.89). Within the LBD group, plasma NfL was not associated with any clinical scores, controlling for age and sex (MMSE: β=-0.00, p=.98; MOCA: β=0.01, p=.87; Cognitive composite score: β=-0.01, p=.47; UPDRS-total: β=-0.02, p=.97; UPDRS-III: β=0.05, p=.83).

### **Fixel-based analysis**

#### **Group Differences in FDC**

FDC was reduced for people with LBD relative to PD-low risk in the genu of the corpus callosum, bilaterally in the anterior corona radiata, and bilaterally in the sagittal stratum and posterior thalamic radiation. FDC was greater in the LBD group relative to PD-low risk regions including the body and splenium of the corpus callosum, bilateral posterior limb of internal capsule, bilateral posterior and superior corona radiata, cingulum, right superior longitudinal fasciculus and bilateral cerebral peduncle (Figure 2C). No differences were observed in FDC between DLB and PDD. No differences in FDC were observed between LBD and control groups.

**Association with plasma concentrations and clinical variables in LBD**

Higher concentrations of plasma p-tau217 were associated with reduced FC in people with LBD risk in the left superior longitudinal fasciculus (Supplementary Figure 4). Likewise, higher concentrations of plasma NfL were associated with reduced FC in the left superior longitudinal fasciculus. Neither plasma measure was associated with FD in people with LBD.

No significant associations were observed between fixel based measures and clinical scores, when using a FWE corrected (p<.05) threshold for significance. Due to the smaller sample size of the LBD group, these analyses may be underpowered to detect any potential differences. Therefore, we also examined associations using an uncorrected threshold (p<.001). This showed significant associations were observed between FC and cognition in people with LBD (Supplementary Figure 7). Lower cognitive scores were associated with lower FC in the sagittal stratum; additionally lower MOCA and composite cognitive score were associated with FC in the left anterior corona radiata and external capsule. Lower cognitive scores were also associated with higher FD in the posterior thalamic radiation and left superior longitudinal fasciculus, as well as with lower FD in the anterior corona radiata and superior longitudinal fasciculus.

Significant associations were also observed between UPDRS and fixel-based metrics when using an uncorrected threshold (Supplementary Figure 8). Higher UPDRS-III scores were associated with lower FC in the posterior limb of internal capsule and middle cerebellar peduncle; and with lower FD in the anterior corona radiata, cingulum, superior longitudinal fasciculus and posterior thalamic radiation. Higher UPDRS-total scores were associated with lower FC in the external capsule, anterior corona radiata and uncinate fasciculus. Higher UPDRS-total scores were associated with lower FD in the body of corpus callosum and posterior thalamic radiation, and higher FD in the anterior limb of internal capsule and posterior thalamic radiation.

## Supplementary Figures

**Supplementary Figure 1. Association between time taken to complete Stroop colour naming and predicted time based on time taken to complete half-Stroop colour naming.**


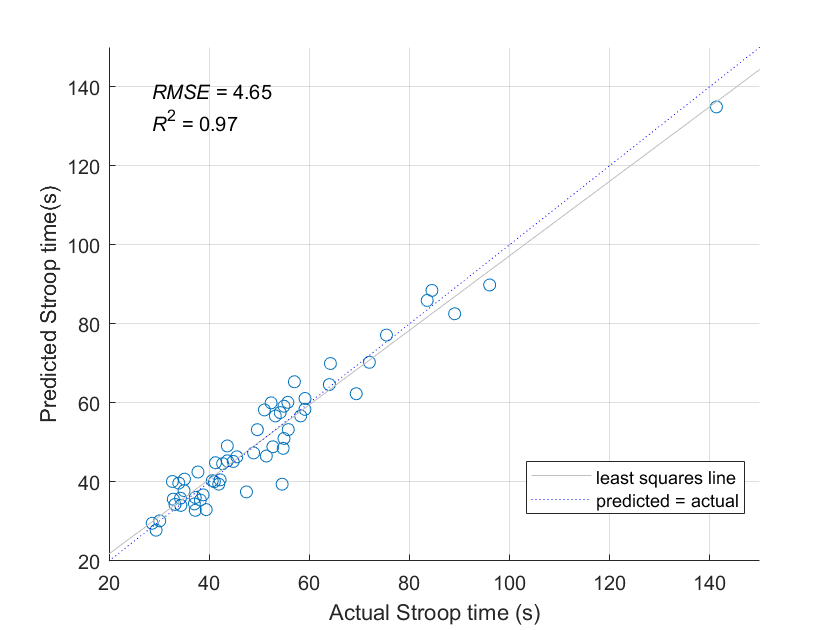


**Supplementary Figure 2. Plasma p-tau217 and NfL group associations with cognitive scores. A**) Association of plasma p-tau217 with MMSE score; **B**) Association of plasma p-tau217 with composite cognitive score; **C**) Association of plasma NfL with MMSE score; **D**) Association of plasma NfL with composite cognitive score. MMSE=Mini Mental-State Examination


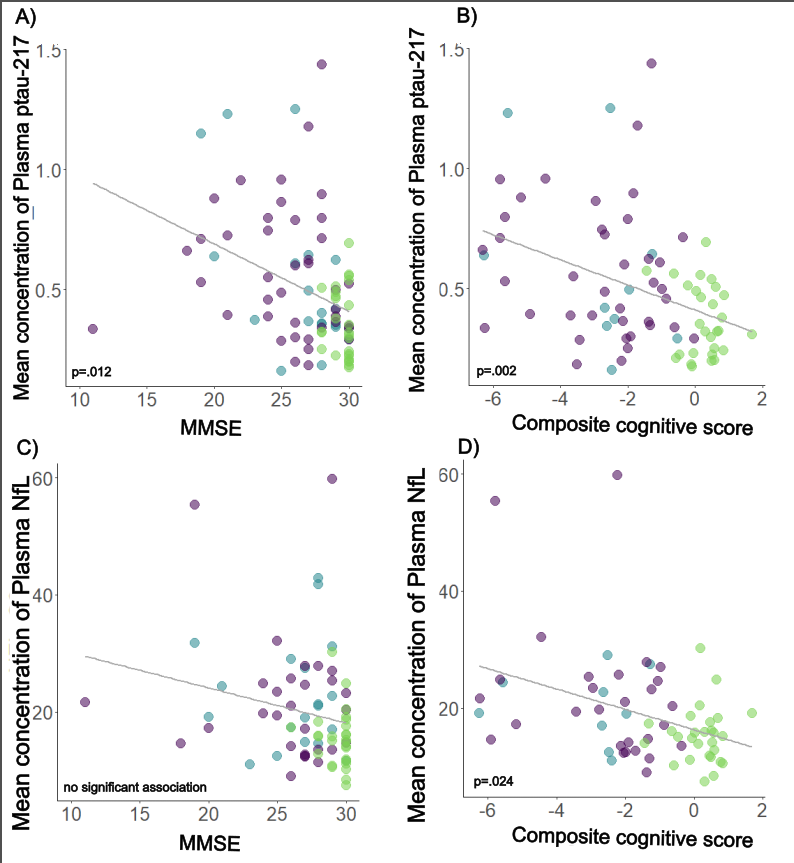


**Supplementary Figure 3. Associations of plasma measures with cognition within LBD. A**) Association of plasma p-tau217 with MMSE, MOCA and cognitive composite score within LBD; **B**) Association plasma NfL with MMSE, MOCA and cognitive composite score within LBD. MMSE=Mini Mental-State Examination, MoCA = Montreal Cognitive Assessment

**
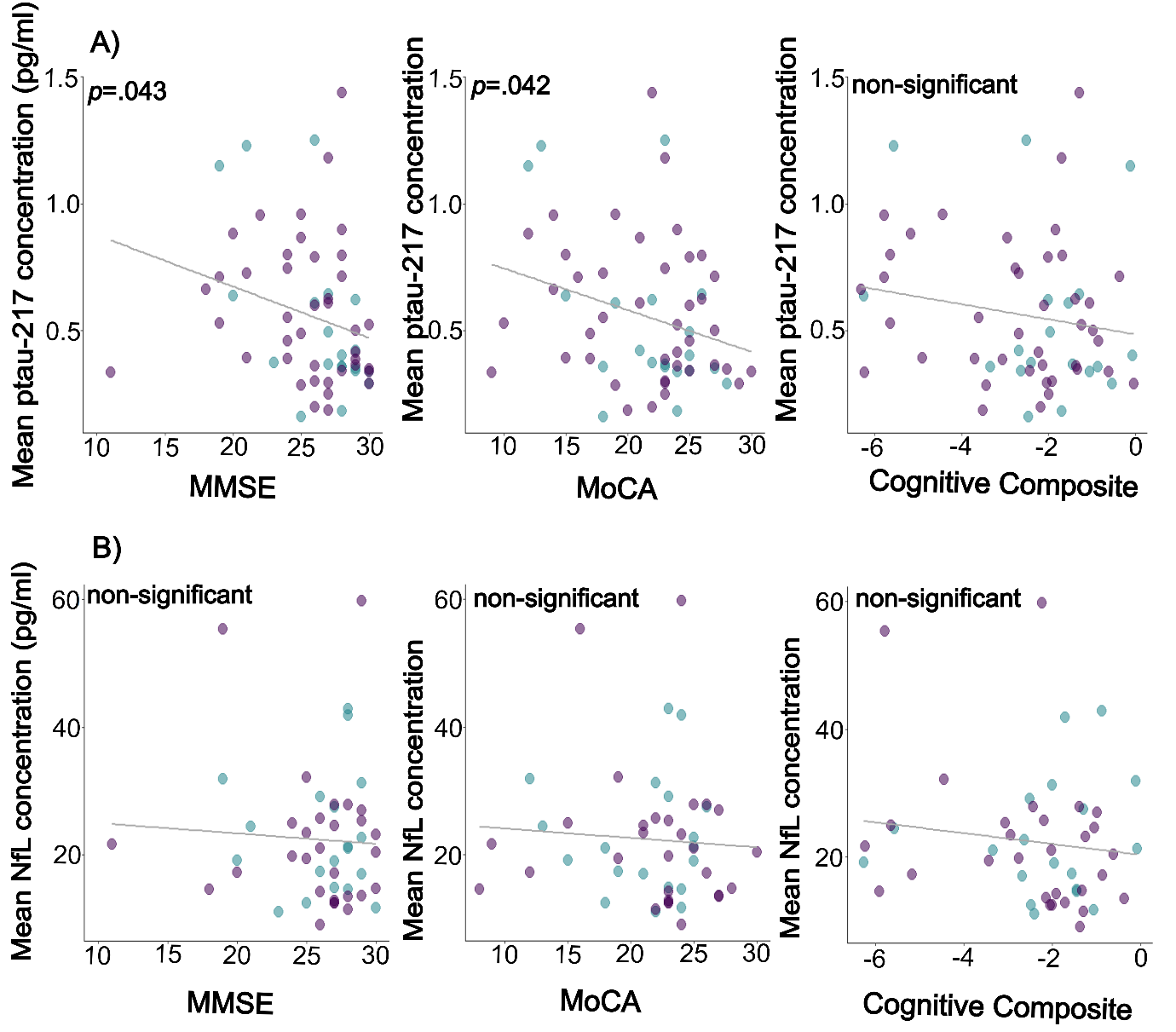
**

**Supplementary Figure 4. Associations between fixel-based fibre cross section and plasma measures in LBD. A**) Association of higher plasma NfL with lower fibre cross section, **B**) Association of higher plasma p-tau217 with lower fibre cross section. All results are displayed as fixels that were significantly associated with cognitive measures (*p* _FWE_<.05). Streamlines are displayed on the group white matter template and coloured by absolute effect (colour bars).

**
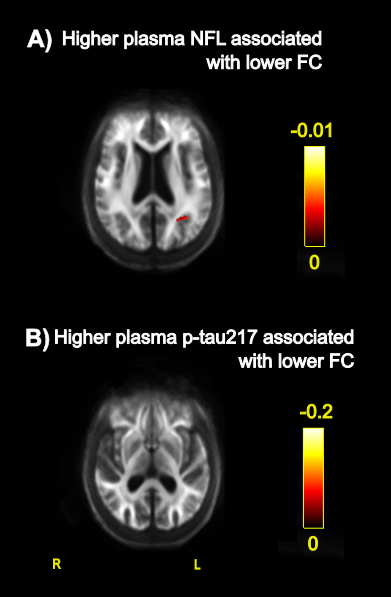
**

**Supplementary Figure 5. Associations between fixel-based fibre density and cognitive scores across all LBD and PD-low risk patients.** Association of **A**) higher MoCA score with reduced fibre density, **B**) higher composite cognitive score with higher fibre density. All results are displayed as streamlines corresponding to fixels that were significantly associated with cognitive measures (*p* _FWE_<.05). Streamlines are displayed on the group white matter template and coloured by absolute effect (colour bars).


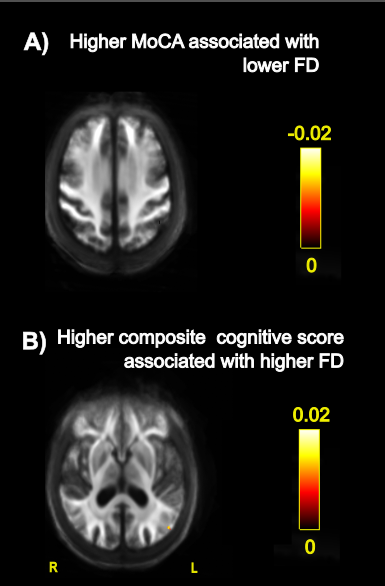


**Supplementary Figure 6. Associations between fixel-based fibre cross-section and UPDRS-III score across all LBD and PD-low risk patients.** All results are displayed as streamlines corresponding to fixels that were significantly associated with cognitive measures (*p* _FWE_<.05). Streamlines are displayed on the group white matter template and coloured by absolute effect (colour bars).


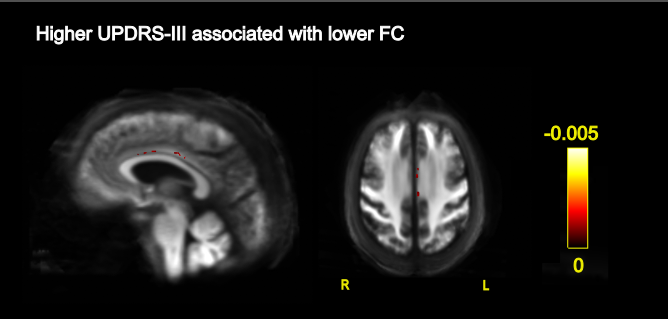


**Supplementary Figure 7. Associations between cognitive scores and fixel-based fibre cross section and fibre density.** Association of higher MMSE score with **A)** higher fibre cross-section, **B)** higher fibre density. Association of higher MOCA score with **C**) higher fibre cross-section, **D)** higher and lowed fibre density. Association of higher composite cognitive score with **E**) higher fibre cross-section, **F)** higher fibre density. All results are displayed fixels that were significantly associated with cognitive measures (*p* _uncorrected_<.001). Streamlines are displayed on the group white matter template and coloured by absolute effect (colour bars).


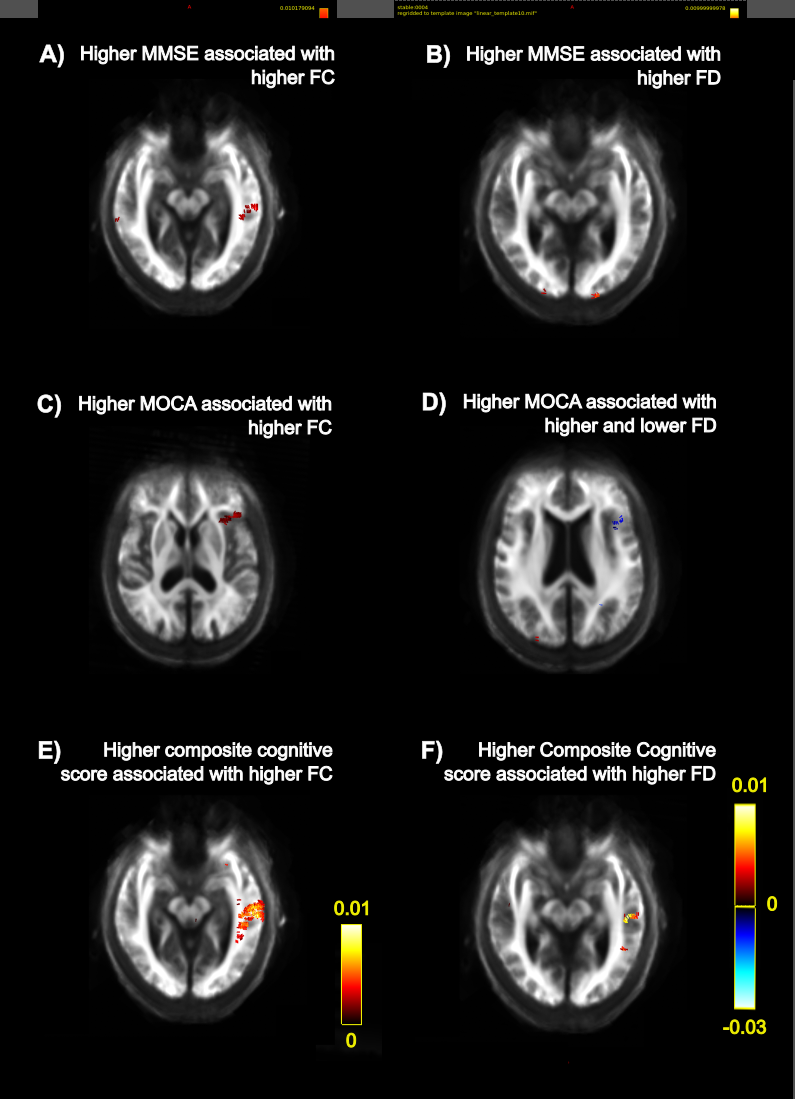


**Supplementary Figure 8. Associations between UPDRS scores and fixel-based fibre cross section and fibre density.** Association of higher UPDRS-III score with **A)** lower fibre cross-section, **B)** lower and higher fibre density. Association of higher UPDRS-total score with **C**) lower fibre cross-section, **D)** lower fibre density. All results are displayed fixels that were significantly associated with cognitive measures (*p* _uncorrected_<.001). Streamlines are displayed on the group white matter template and coloured by absolute effect (colour bars).


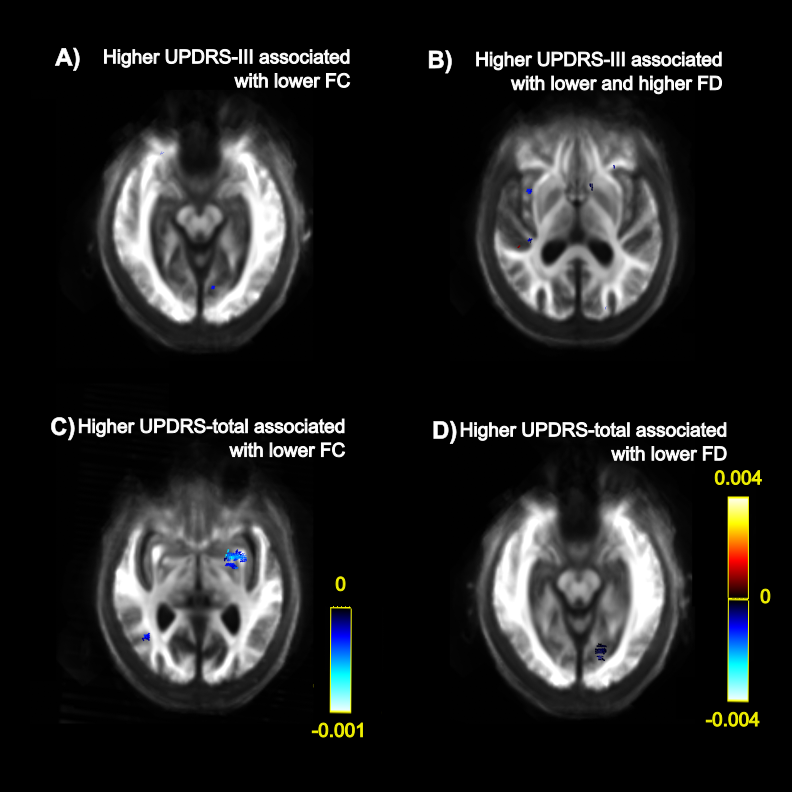

Supplement: Supplementary file 1 — Supporting Information [file ALZ-21-e70274-s001.docx]
